# Thyroid hormone as a temporal switch in mouse development

**DOI:** 10.1530/ETJ-22-0225

**Published:** 2023-03-10

**Authors:** Juan Ren, Frédéric Flamant

**Affiliations:** 1 ENS de Lyon, INRAE, CNRS, Institut de Génomique Fonctionnelle de Lyon, Lyon, France

**Keywords:** thyroid hormone, mouse, postnatal development, metamorphosis

## Abstract

Thyroid hormones are known to trigger metamorphosis in an amphibian. This review discusses the hypothesis according to which they act in a similar manner to synchronize the post-natal development of mice, using brain, brown adipose tissue, and heart as examples.

Thyroid hormones (THs; including T3, 3, 3′, 5-triiodo-l-thyronine and its less active precursor T4 or thyroxine) exert a broad influence on the development of vertebrate species. The possibility that the developmental function of TH in mammals is similar to that in amphibians and fish metamorphosis has been discussed on several occasions (1, 2, 3). This review takes advantage of the available genetic toolbox to reconsider the hypothesis that mouse postnatal development represents a metamorphosis-like process.

## Similarity between amphibian metamorphosis and mouse postnatal development

THs have originally been discovered as signals that trigger amphibian tadpole metamorphosis (4). During these crucial developmental stages, a number of dramatic changes take place in the body plan, which have been extensively studied in the anuran *Xenopus laevis*: gills disappear, hindlimbs grow while the tail regresses, and the intestine and the brain are deeply remodeled. The position of the eyes and the retina projections are also modified. Early exposure of tadpoles to TH precociously induces metamorphosis, whereas TH suppression delays it extensively. Therefore, it appears that THs do not represent an instructive signal that is able to impose a complete change in the developmental trajectory, but mainly a permissive signal that acts as a timer of metamorphosis. Although other hormones, notably cortisosterone (5), also influence the transition, TH function is essential, because a proper timing of events during metamorphosis is vital.

TRα and TRβ are the nuclear receptors of T3, respectively, encoded by the *Thra* and* Thrb* genes, which are already expressed in tadpoles before metamorphosis. They display favorable properties converting the elevation of TH into a sharp developmental transition. First, they act as DNA-binding transcription repressors in the absence of ligands and quickly switch into transcription activators upon T3 binding (6). Furthermore, this abrupt switch is amplified by the fact that the expression of* Thrb*, and to a lesser extent of *Thra*,* is* upregulated by T3 (7).

**Figure 1:**
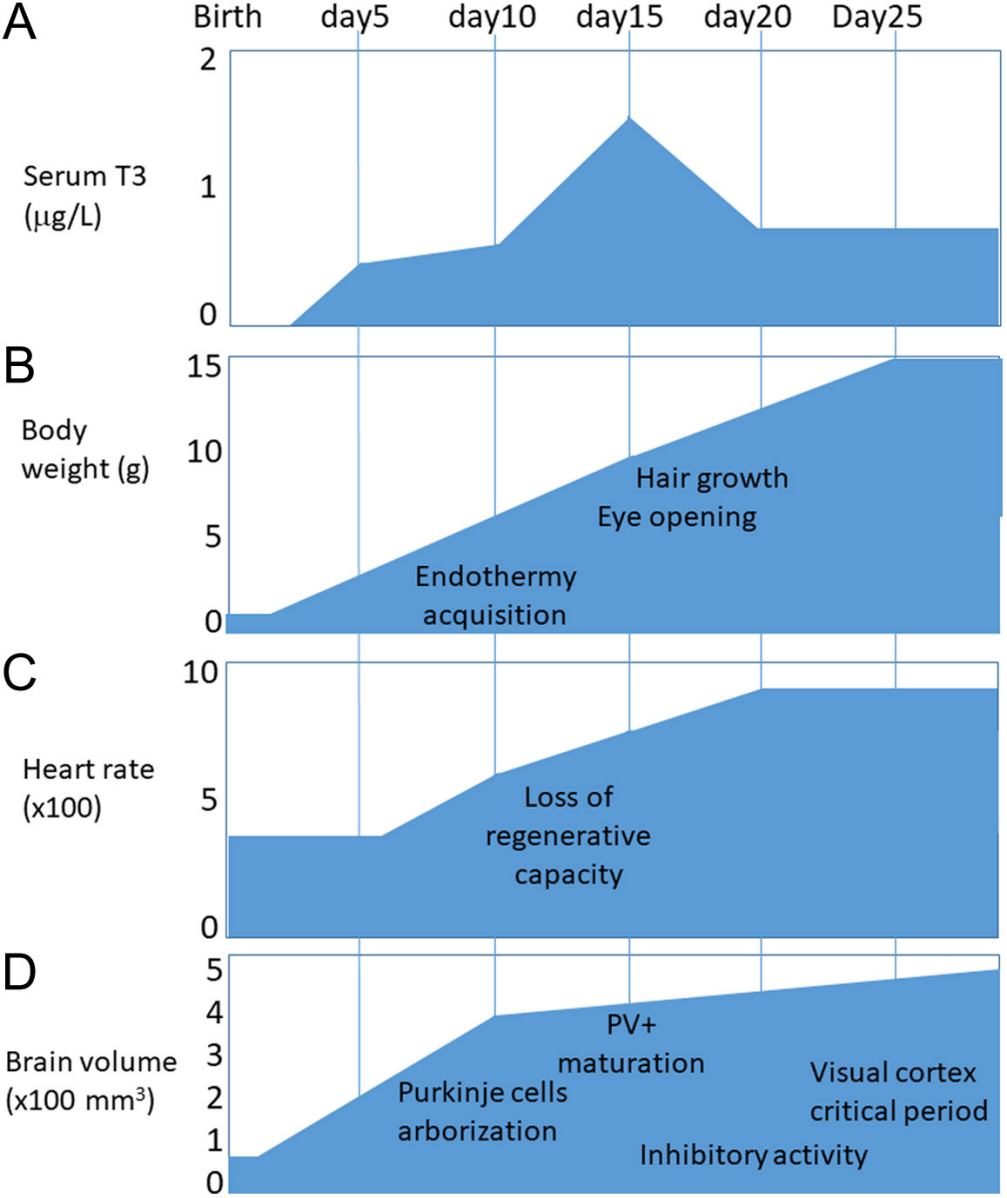
Postnatal maturation processes in mice. (A) Evolution of the T3 concentration in serum (8). (B) Eye opening occurs at around postnatal day 13 (PND13). Endothermy is achieved progressively before PND15. The anagen phase of hair growth is at PND17. Weaning becomes possible on PND21. (C) Cardiomyocytes lose their regenerative capacity (18) when heart rate (84) and oxidative capacities of ventricular fibers (85) increase during the second postnatal week. (D) The brain grows faster than the entire body. Forebrain regions, such as the olfactory bulbs and cortex, grow earlier than cerebellum (86). In the cerebellum, the arborization of Purkinje cells progresses between PND7 and PND21. In cortical basket cells, also known as fast-spiking interneurons, a major change in ions channels content occurs around PND15 (42). They express the parvalbumin marker and exert an inhibitory activity which has a pivotal role in the elaboration of the neuronal circuits (87). A phase of synaptogenesis is followed by the critical period of heightened plasticity (43). The peak of synaptic plasticity occurs at different times in different cortex areas, from PND12 in the somatosensory cortex to PND28 in the visual cortex. At the closure of the critical period, basket cells produce the perineuronal net which stabilizes their synapses (44). The inhibitory activity of GABAergic neurons finally balances the excitatory activity of glutamatergic neurons (88). NB: The milestones of postnatal development vary slightly depending on the mouse strain.

Mice are called altricial because they are underdeveloped at the time of birth and rely heavily on maternal care. During the first 3 weeks of postnatal life, major developmental processes are at work (Fig. 1). In particular, the brain undergoes important maturation processes during the postnatal period, which are equivalent to those occurring during the second trimester of human gestation. While the mouse fetal development is primarily dependent on maternal-derived TH, the mouse thyroid gland becomes functional before birth and provides all the necessary TH after birth. Although less pronounced than during amphibian metamorphosis, a marked increase in both serum T4 and T3 levels is observed during the first 2 postnatal weeks (8), after which TH levels decrease and become stable in serum. The importance of TH in mouse postnatal development has been demonstrated by knocking out the* Pax8* gene (9, 10). This genetic ablation of the thyroid gland causes lethality, occurring during the second and third postnatal weeks unless the mice are rescued by TH treatment.

The mammalian *Thra* gene encodes several non-receptor proteins (11) and the TRα1 receptor, which is nearly ubiquitous. The *Thrb* encodes TRβ1 and TRβ2. Whereas TRβ2 mRNA is found in only a few cell types, TRβ1 mRNA is broadly distributed. TRβ1 is abundant in the liver, heart, and several brain areas where it appears at late stages of development (12). Their protein abundance is poorly documented, but the recent development of mouse models with tagged *Thra* or* Thrb* alleles revealed surprising discrepancies between mRNA and protein levels. In particular, the non-receptor protein TRα2 is more abundant than TRα1 in brain, and TRβ1 is less abundant than predicted in several organs (13). Overall, most if not all mammalian cells have the ability to respond to T3 throughout development. However, in contrast to the amphibian genes, the response of the mouse *Thrb* gene expression to T3 is modest, and restricted to a few cell types, while *Thra* gene tends to be downregulated (14). Therefore, the T3 response in mammals is unlikely to be amplified by a positive feedback loop resulting in receptors accumulation.

In the following, we will consider major transitions in which TH have been demonstrated to be involved in mouse postnatal development. These are only examples, and we do not intend to cover the large body of literature related to the influence of TH. In each case, the main difficulty consists in distinguishing between the direct consequences of TH stimulation, also called cell-autonomous response, and the indirect influences, which are secondary to the local or systemic changes caused by TH. In the chosen examples, this discrimination has been addressed by using Cre/loxP recombination to selectively alter the T3 response in selected cell types.

## From ectotherm to endotherm

Mouse pups only progressively gain the ability to maintain their body temperature, notably by developing the brown adipose tissue which is specialized in adaptive thermogenesis. Although a small number of brown adipocytes are present at birth, the cell proliferation in this tissue peaks at postnatal day 8 (PND8) (15). However, while the brown adipocytes already contain the UCP1 uncoupling protein, which is thought to be at the heart of the thermogenic process, they do not express the genes encoding key lipogenic enzymes before PND15. Therefore, the brown adipose tissue of juvenile mice is unable to fuel thermogenesis autonomously, as it does in adults, but rather oxidizes fatty acids coming from the maternal milk. Furthermore, the neuronal circuits required to sense cold and trigger thermogenesis only become functional at PND15 (16).

As THs are known to promote several thermogenic processes, including adaptive thermogenesis in brown adipose tissue, one would expect a direct link between TH and the maturation of the thermogenic capacity of pups. Although to our knowledge, the juvenile phase has not been specifically addressed, selective deletion of TRα1 in brown adipocyte progenitors alters their capacity to produce differentiated cells in adults (17). Consequently, adult mice lose the capacity to expand their brown adipose tissue after prolonged T3 stimulation. It is therefore likely that the original development of the brown adipose tissue is sensitive to the transient postnatal increase in T3 level.

## The loss of regenerative capacity of the heart

Mice are born with the capacity to regenerate the cardiac muscle after injury, but lose this capacity within 2 weeks after birth. In this process, immature cycling mono-nucleated cells differentiate into bi-nucleated cells, which have a high capacity for oxidative phosphorylation but are unable to proliferate. Exposure to goitrogen propyl-thio-uracyl or the TRα1 antagonist NH-3 greatly delays this transition (18).

When the dominant-negative mutation TRα1^L400R^ is expressed only in cardiomyocytes to block their capacity to respond to T3, a persistent proliferation of cardiomyocytes is observed. The heart size increases in pathological proportions, and its regenerative capacity, which manifests after an infarction, persists at the adult stage. Therefore, the influence of T3 on the loss of regeneration capacity is cell-autonomous. Transcriptome analysis detects changes in gene expression caused by TRα1^L400R^ expression in cardiomyocytes, while ChIP-Seq analysis pinpoints genes whose regulatory sequences are occupied by TRα1. This combined strategy allows to identify a set of genes whose transcription is very likely to be activated by the liganded TRα1 in the postnatal heart and to promote the loss of regeneration capacity. A number of them encode important enzymes and regulators of oxidative phosphorylation in mitochondria.

A survey of many vertebrate species suggests that the loss of heart regenerative capacity is a trade-off for the acquisition of endothermy during evolution. Endothermy is indeed energy demanding and imposes a high contraction capacity on the myocardium, to ensure a proper oxygen supply to tissues. In mammals, cardiomyocyte metabolism switches to ATP-producing processes. A unifying hypothesis would be T3 reprograms cardiomyocytes metabolism, which switches from nucleotide synthesis to ATP production to face a high-energy demand during mammalian development. Accordingly, zebrafishes, which are ectotherm, maintain the regenerative capacity of their heart at the adult stage (19), unless they are treated with an excess of T3 (18). Interestingly, a metabolic switch from mitochondrial oxidative phosphorylation to glycolysis, associated with a remodeling of the extracellular matrix glycosylation, accompanies the zebrafish heart regeneration (20). However, the heart of *Xenopus laevis*, which is also an ectotherm species, loses its regeneration capacity under the influence of T3 and the transition takes place during metamorphosis (21). Therefore, the tempting link between the function of T3 in development and in thermogenesis requires further investigation.

## Loss of axonal regenerative capacity in Purkinje cells

The evolutionary loss of regenerative competence in mammals, and its maintenance in zebrafish, also applies to neurons. This manifests either as injury-induced neurogenesis and axonal regeneration after axons severing. Here again, a developmental transition has been observed in mice. Notably, the axons of Purkinje cells, in the cerebellum, possess the capacity to regrow at early postnatal stages. This regeneration capacity disappears within 1 week after birth, whether when the cells stay in the cerebellum or if cerebellum slices are cultured *in vitro* (22). In general, T3 exerts a cell-autonomous influence on the morphological maturation of these neurons, which takes place after birth (23, 24). The typical arborization of Purkinje cells is impaired by either TRα1 or TRβ1 mutations (25, 26). Therefore, while T3 prevents axonal regrowth, it also favors dendritic growth. The most likely explanation is that the link to T3 is indirect and that both the loss of regenerative capacity and the dendritic arborization reflect the terminal maturation of the Purkinje cells.

The capacity of the Purkinje cell axons to regenerate can be evidenced either by performing *in vivo* or *in vitro* axotomy. T3 has a clear effect on the timing of this transition, accelerating the loss of regenerative capacity both *in vivo* and *in vitro*. Accordingly, the dominant-negative receptor TRα1^L400R^ delays the loss of regeneration capacity (22). In some other neurons, axon myelination has been shown to be responsible for the loss of regeneration capacity. The myelination in itself is a T3-dependent process, as T3 activates the differentiation of oligodendrocytes, the cells which create myelin sheaths (27). This raises the possibility of an indirect effect of T3 of Purkinje cells, mediated by oligodendrocytes. However, this hypothesis was ruled out by expressing TRα1^L400R^ only in Purkinje cells, using a lentivirus vector (22). Although the molecular details remain unknown, gain and loss of function experiments demonstrate that the Klf9 transcription factor is an important intermediate of T3 action. As in many other cell types, the expression of the* Klf9* gene is activated by the T3-bound TRα1 receptor in Purkinje cells. The Klf9 transcription factor then modifies the expression of other genes, and the cells lose the capacity to regenerate their axon. In general, loss of regeneration capacity appears to be one of the T3-dependent processes during Purkinje cell maturation. Overall, the possible involvement of T3-signaling in the developmental and evolutionary loss of regenerative capacity remains one hypothesis among several others (28).

## Maturation of the GABAergic interneurons of the cortex and the critical period

Development of the cerebral cortex is an extremely complex process. Neurons migrate and form a layered structure and then establish synapses to finally generate functional circuits. Congenital hypothyroidism in rodents leads to less defined cortical layers, defective neuronal migration and altered circuitry (29) causing irreversible cognitive defects (30). Virtually all cortical cell types are affected by TH deficiency (31). However, because neurotrophins secretion is altered (32), it is unclear whether all cell lineages display an autonomous response to T3. The alternative would be that a small fraction of cells are sensitive to T3 and then orchestrate a network of cellular interactions. This can be mediated by direct cell contacts and the secretion of diffusible factors, as previously demonstrated in the cerebellum (33).

Cortical cells for which the influence of T3 has been shown to be cell-autonomous are the GABAergic inhibitory interneurons. The expression of TRα1^L400R^ restricted to this lineage causes lethal epileptic seizures (34). The GABAergic neuronal population includes at least 12 different cell types defined by their morphology, position, connections, immunostaining, and gene expression (35). Whether all 60 types and subtypes are equally sensitive to T3 remains unclear. However, a selective alteration has been observed for the maturation of parvalbumin-expressing (Pv+) cells, which normally represent 40% of the cortical GABAergic population. A large fraction of Pv+ cells are called basket cells based on morphology, or fast-spiking interneurons based on electrophysiological properties (36). Hypothyroidism and mutations altering TH transport across the blood–brain barrier (37) greatly reduce the density of Pv+ cells in the cortex at PND15 (38, 39). The same defect can be observed in mice which express TRα1^L400R^ (34) or TRα1^R384C^ , another mutation which greatly reduces the affinity of the receptor for T3 (40). Besides, patch-clamp recording of TRα1^R384C/+^ cortical slices revealed a dramatic reduction in the number of fast-spiking interneurons (40). As TRα1^R384C^ has a residual sensitivity to T3, it is less detrimental than expressing TRα1^L400R^ that is often lethal. A slow recovery of Pv+ cells is observed in TRα1^R384C/+^ mice, but not in the few surviving TRα1^L400R/+^ mice. This suggests that T3 not only defines the timing for the maturation of these cells but also maintains their function in the adult brain.

Basket cells produce spontaneous γ-oscillations soon after birth, which synchronize the early electrical activity of cortical networks (41). They undergo an extensive functional and transcriptional maturation process between PND10 and PND40, with a major change in ion channels content around PND15 (42). They ultimately produce a specific type of extracellular matrix called the perineuronal net, which stabilizes synapses. The assembly of the perineuronal net marks the end of the so-called critical period of high synaptic plasticity (43, 44). After this, experience-dependent elaboration of novel neuronal circuits becomes almost impossible. The elaboration of the perineuronal net is altered in mice expressing TRα1^L400R^ in GABAergic neurons (34). Therefore, it is tempting to conclude that, by ensuring the proper timing of basket cells maturation, T3 provides a temporal signal, which defines the end of the critical period (45). It would be of interest to address if these conclusions also apply to the filial imprinting of birds. Similarly, this phenomenon is restricted to a sensitive period defined by T3 (46) and involves GABAergic neurons (47).

## Desynchronizing the synchronization

The earlier examples indicate that during the postnatal and preweaning period, T3 acts in a cell-autonomous manner to promote the terminal maturation of distinct cell types: the cardiomyocytes, the Purkinje cells of the cerebellum, and the cortical basket cells. This conclusion probably also applies to at least bone chondrocytes (48) and spleen erythrocytes progenitors (49). On the other hand, there are cell types that respond in a non-cell autonomous manner to T3 during development. For example, the response to T3 of the granule cells progenitors of the cerebellum is only secondary to the T3-induced secretion of diffusible signals by the neighboring Purkinje cells (25). Similarly, the oligodendrocyte precursor cells, responsible for the initial postnatal myelination of axons, are not sensitive to the restricted expression of TRα1^L400R^ (50, 51). Therefore, T3-responsive cells amplify the influence of T3, secreting growth factors and neurotrophins that influence their microenvironment (52, 53, 54).

The possibility that T3 is a temporal signal orchestrating directly or indirectly the multiple changes that take place after birth in mice justifies the comparison with amphibian metamorphosis. In that respect, it is striking that the heart regenerative capacity is lost in both models. However, under this hypothesis, all T3-dependent maturation processes should be initiated when the circulating level of T3 is at its maximum, in tadpoles or juvenile mice, which is not precisely the case. In particular, the critical period of heightened plasticity mentioned above is restricted to a temporal window, which varies according to brain areas (43). Thus, in addition to the circulating level of T4 and T3, one should consider the local level of signaling, whose dynamic evolution after birth might be different in different areas and cell types. Reporter mice indeed reveal a complex and dynamic pattern of T3 signaling in brain (55).

The most obvious way to modify the local concentration of T3 and generate different peaks of T3 at different times would be deiodination. Several studies illustrate this possibility for inner ear maturation and the onset of hearing. In the prenatal cochlea, type 3 deiodinase catabolizes both T4 and T3, lowering the T3 content of the perilymph (56). After birth, this enzyme is replaced by type 2 deiodinase that quickly increases the local concentration of T3 by converting T4 into T3, which ensures a timely differentiation of mechanosensory hair cells within the cochlea (57). Therefore, while T3 peaks in the serum at PND15, the maximum T3 concentration in the cochlea is achieved at PND7. If supplied in excess at birth, T3 advances the overall program of cochlear maturation by several days, causing deafness in adult mice (58). The dynamic changes in deiodinases activity during postnatal development provoke similar uncoupling between the circulating and tissue levels of T3 in other tissues, including the intestine and liver (59, 60).

An alternative mechanism for the local modulation of T3 signaling relies on transporters. Several transporters ensure the transport of TH across the placenta, blood–testis and brain–blood barriers, and their transfer to the cell nucleus. While human patients with *Mct8* mutations (alias *Scl16a2)* suffer from deep brain hypothyroidism, this is not the case for *Mct8* knock-out mice (61). In these mice, a moderate T4 deficit is compensated by increased type-2 deiodination (62) maintaining sufficient T3 in the postnatal brain (37). However, overt brain hypothyroidism appears when the *Mct8* knock-out is combined with a second knock-out to eliminate either another transporter *(Oatp1c1/Slc01c1)* (63) or type 2 deiodinase (64).

## Future directions and unsolved problems

The fruitful cross-species comparison which led us during this analysis suggests several fruitful avenues for future research and should not be limited to *Xenopus laevis* and mice. In particular, zebrafish is a popular model which introduces a useful complement as it is ectotherm, has a high regeneration capacity, and does not undergo an obvious metamorphosis. This model has not yet been fully exploited and provides attractive models to analyze the developmental function of T3, notably in bones (65) skin (66, 67) retina (68, 69), and lateral line, which is a sensory organ which function is comparable to the one of the inner ear (70).

The reporter mice that allow to visualize the heterogeneity of T3 signaling in the fetus and in the postnatal brain (55) have been crossed to several knock-out mice in order to identify the origin of this heterogeneity. Surprisingly, the elimination of type 2 deiodinase had no influence on the temporal and spatial expression of the reporter transgene (unpublished data). Only after several months did the absence of type 3 deiodinase cause the accumulation of T3 in specific areas of the adult brain (71). The knock-out of the *Mct8/Scl16a2* gene also failed to modify the perinatal expression pattern of the reporter transgene (unpublished data). Finally, the reporter mice were crossed to mice carrying a mutation of the *Hairless* gene, which in many cell types is among the most T3-responsive genes. *Hairless* encodes a protein which has been proposed to be a TR corepressor (72), but its function remains unclear. The loss of function of *Hairless* did not alter the expression of the reporter transgene, either (unpublished data). Although there are technical limitations to this reporter system, which relies on the *in situ* detection of β-galactosidase activity, these observations suggest that an important piece of information is missing, which would explain why T3 is not evenly distributed in mouse tissues during the fetal and postnatal development. Besides, a variety of TH transporters, whose functions are not fully documented, remain to be tested (73). Another interesting candidate is μ-crystallin (74), an enzyme encoded by the *Crym* gene (75), which is abundant in some astrocytes (76) and maintains the intracellular pool of TH (77). *Crym* gene overexpression in mouse muscles causes a local accumulation of T3 and favors lipids β-oxydation (78).

The reason why some cell types are more sensitive than others to T3 stimulation remains unexplained. This sensitivity can be related to the abundance of receptors in a certain cell type. However, this abundance is very difficult to measure directly (13) and alternative explanations need to be explored. In particular, many proteins interact with TRα1 and TRβ1 (79). These proteins shuttle the receptors between the nucleus and cytoplasm (80), introduce post-translational modifications (81, 82), or act as transcription cofactors (83). Finally, in most cell types, the lack of transcriptome and cistrome information hinders the identification of target genes that mediate the functions of receptors.

## Declaration of interest

The authors declare that there is no conflict of interest that could be perceived as prejudicing the impartiality of this review.

## Funding

Research in our laboratory is funded by European Union's Horizon 2020 research and innovation program under grant agreement 825753 (ERGO) and Agence Nationale de la Recherche (ANR22-CE14-0026-01).
